# Multiple sclerosis reduces synchrony of the magnocellular pathway

**DOI:** 10.1371/journal.pone.0255324

**Published:** 2021-08-26

**Authors:** Masoud Seraji, Maryam Mohebbi, Amirhossein Safari, Bart Krekelberg

**Affiliations:** 1 Center for Molecular and Behavioral Neuroscience, Rutgers University, Newark, New Jersey, United States of America; 2 Behavioral and Neural Sciences Graduate Program, Rutgers University, Newark, New Jersey, United States of America; 3 School of Electrical Engineering, K.N.Toosi University of Technology, Tehran, Iran; University of New England, AUSTRALIA

## Abstract

Multiple Sclerosis (MS) is an autoimmune demyelinating disease that damages the insulation of nerve cell fibers in the brain and spinal cord. In the visual system, this demyelination results in a robust delay of visually evoked potentials (VEPs), even in the absence of overt clinical symptoms such as blurred vision. VEPs, therefore, offer an avenue for early diagnosis, monitoring disease progression, and, potentially, insight into the differential impairment of specific pathways. A primary hypothesis has been that visual stimuli driving the magno-, parvo-, and konio-cellular pathways should lead to differential effects because these pathways differ considerably in terms of myelination. Experimental tests of this hypothesis, however, have led to conflicting results. Some groups reported larger latency effects for chromatic stimuli, while others found equivalent effects across stimulus types. We reasoned that this lack of pathway specificity could, at least in part, be attributed to the relatively coarse measure of pathway impairment afforded by the latency of a VEP. We hypothesized that network synchrony could offer a more sensitive test of pathway impairments. To test this hypothesis, we analyzed the synchrony of occipital electroencephalography (EEG) signals during the presentation of visual stimuli designed to bias activity to one of the three pathways. Specifically, we quantified synchrony in the occipital EEG using two graph-theoretic measures of functional connectivity: the characteristic path length (L; a measure of long-range connectivity) and the clustering coefficient (CC; a measure of short-range connectivity). Our main finding was that L and CC were both smaller in the MS group than in controls. Notably, this change in functional connectivity was limited to the magnocellular pathway. The effect sizes (Hedge’s g) were 0.89 (L) and 1.26 (CC) measured with magno stimuli. Together, L and CC define the small-world nature of a network, and our finding can be summarized as a reduction in the small-worldness of the magnocellular network. We speculate that the reduced efficiency of information transfer associated with a reduction in small-worldness could underlie visual deficits in MS. Relating these measures to differential diagnoses and disease progression is an important avenue for future work.

## Introduction

Multiple Sclerosis (MS) is an autoimmune disease that results in both demyelination and axonal injury. Neuronal dysfunction or destruction, the increase in atrophy, and the growth of axonal loss are all associated with a variety of disabilities [1]. As the disease progresses, patients may experience tiredness [2], limb weakness [3], alteration of upper extremity fine motor coordination [4], loss of sensation and cognitive dysfunction [5, 6], speech [7], and visual disorders [8]. In fact, visual disorders are among the most common and early symptoms of demyelinating diseases [9, 10].

Many studies have examined the effect of MS on vision, starting with acuity tests such as the high-contrast Snellen letter test and the Landolt C test. These tests, however, are not reliable diagnostic instruments for MS. Notably, some patients achieve 20/20 scores for visual acuity on the Snellen test, yet they report that their vision is imperfect in one eye or both. This may be explained by the fact that tests such as the Snellen eye chart focus on fine, high-contrast detail, while MS affects visual sensitivity more at lower spatial frequencies [11]. Consistent with this, low-contrast letter tests, i.e., Pelli-Robson contrast sensitivity charts and low-contrast acuity Sloan letter charts, can identify visual dysfunction in MS [10, 12]. The visual properties of stimuli that are most affected match the preferences of the magnocellular visual pathway, which is most sensitive at low contrast and low spatial frequencies.

Based on such behavioral findings and the fact that axon sizes and myelination differ across the magno-, parvo-, and konio-cellular pathways, researchers have hypothesized that MS has a differential effect on the three visual pathways. Studies comparing the latency of visual evoked potentials (VEP), however, have led to conflicting results, with some reporting larger effects for stimuli targeting the parvocellular pathway [13, 14] and others failing to find a difference across pathways [15, 16].

Some of these discrepancies may be due to the limited ability of a visual stimulus to target one visual pathway specifically [17]. On the other hand, the limited specificity could also be caused by the relatively coarse measure of neural activity afforded by the latency of a VEP. Here, we addressed this latter point by analyzing the synchrony of visual responses across occipital electroencephalography (EEG) signals. Recently, such functional connectivity analyses have identified large scale synchrony deficits in several neurological diseases, including MS [18–21]. We hypothesized that this network analysis could provide novel insight into the visual deficits in MS and, without major changes to clinical practice, result in the development of a refined diagnostic tool with increased specificity. To test our hypothesis, we recorded EEG from people living with MS and controls while they viewed visual stimuli designed to bias activity to one of the three visual pathways. From the EEG data, we constructed graphs describing the synchrony among nine occipital channels. Analyzing these graphs revealed that characteristic path length (a measure of long-range connectivity) and clustering coefficient (a measure of local connectivity) were both decreased in the MS group compared to controls. Together, these connectivity changes resulted in a decrease in small-worldness that was specific to the magnocellular pathway.

## Methods

All experimental procedures conformed to the principles of the Declaration of Helsinki and were approved by the Bioethics Committee of Iran University of Medical Science (protocol# IR.IUMS.REC.1395.9406414). All participants took part voluntarily and, after being informed about the study procedures and goals, provided their written consent.

### Subjects

We recruited thirty-seven subjects to participate in these experiments. Twenty were people living with MS, seventeen were age- and sex-matched controls. All participants had a normal or corrected-to-normal vision, with no color-blindness. Patients with a diagnosis of relapsing-remitting MS following the McDonald criteria [22] were recruited from the Iran MS center in Tehran. We rejected the data sets of four patients and one control due to excessive motion artifacts (See Data Preprocessing below). Table 1 shows an overview of the characteristics of the 16 patients (age 39.0 ±7.8 years (mean ± standard deviation)) included in the data analysis. All patients were on Fingolimod or CinnoVex medication. The average age of the 16 controls was 33.7 ± 9.8 years.

**Table 1:** Patients’ information list.

PATIENT	AGE	DISEASE DURATION	GENDER	EDSS
**P1**	43	15	F	4
**P2**	56	11	F	2
**P3**	48	5	F	1.5
**P4**	32	6	F	3
**P5**	29	9	F	2
**P6**	51	14	F	2
**P7**	38	2	F	1.5
**P8**	38	9	F	2
**P9**	40	20	F	2
**P10**	38	12	F	2
**P11**	40	11	M	1
**P12**	38	14	M	4
**P13**	32	1	M	2
**P14**	28	5	M	3
**P15**	44	13	M	2
**P16**	29	4	M	3

The participant’s age and time since diagnosis at the time of the experiment, their gender, and their score on the expanded disability status scale (EDSS)[23]. Most had mild to moderate disability.

### EEG

All EEG recordings took place at the Iran National Brain Mapping Lab (NBML). We used g.HIamp biosignal amplifier (g.tec company) with 32 active electrodes positioned according to the International 10–20 System to record the visually evoked responses. The sampling rate was 512 Hz, the A2 electrode served as a reference electrode, and Fpz was connected to the ground. To avoid introducing artifactual shared components into the connectivity analysis, we did not rereference the data [24].

### Visual stimulus and paradigm

**Fig 1:**
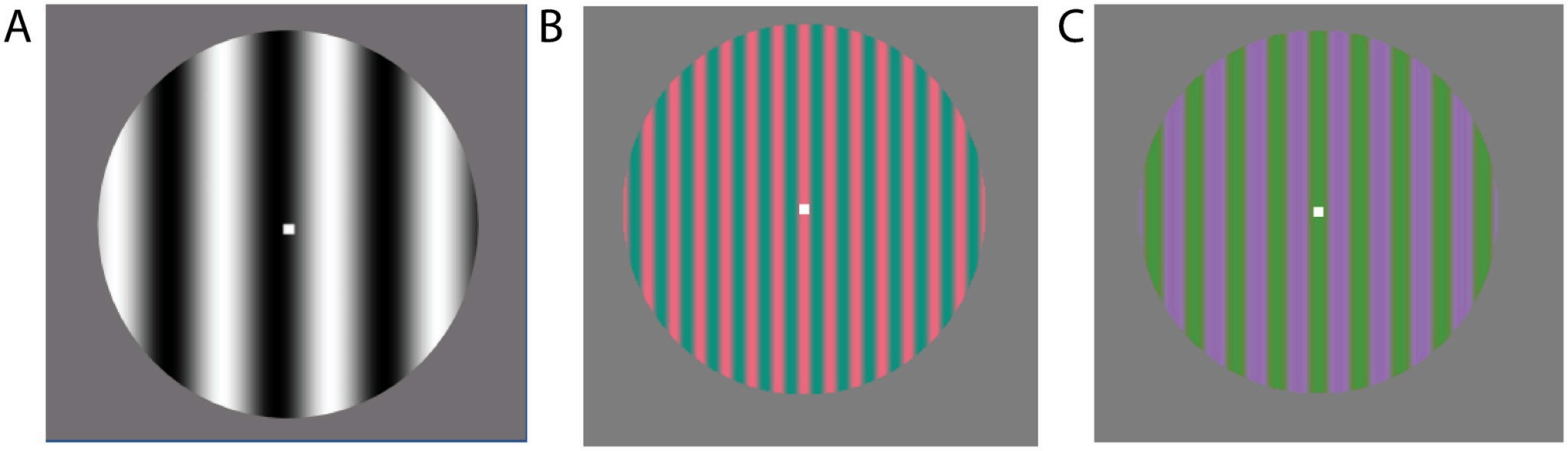
Magno, parvo, and konio stimuli. (A) Magno stimulus (monochrome, low spatial frequency, high temporal frequency, high luminance contrast) (B) Parvo stimulus (red-green color contrast, high spatial frequency, low temporal frequency, low luminance contrast) (C) Konio stimulus (green-purple color contrast, high spatial frequency, low temporal frequency, low luminance contrast).

We used stimuli designed to enhance the response of one pathway compared to the other pathways. All stimuli were sinusoidal gratings with sinusoidal counter phase flicker presented on a neutral gray background. Subjects viewed these stimuli binocularly. All stimuli were presented within a circular aperture with a diameter of 7 degrees of visual angle. A small fixation point was shown throughout the trial at the center of the grating, and subjects were instructed to fixate this point. Although no visual stimulus can truly isolate a specific pathway, visual properties can be used to bias activity to each of the three pathways. For the magno stimulus, we used a grayscale grating with a spatial frequency of 0.5 cycles per degree (cpd), a temporal frequency of 15 Hz, and 100% luminance contrast (Fig 1A). The parvo stimulus was a high color contrast red-green grating with a spatial frequency of 2 cpd, a temporal frequency of 5 Hz, and low luminance contrast (Fig 1B). The konio stimulus was a low luminance contrast, high color contrast yellow-blue grating with a spatial frequency of 1.25 cpd, and a temporal frequency of 10 Hz (Fig 1C). Colors were chosen in DKL color space then transformed to RGB. In each trial, the visual stimulus was presented for 1 second, and the screen was gray during the 2 s intertrial interval [25].

### Data analysis

#### Preprocessing

We applied standard preprocessing procedures in EEGLAB (version 2021) to reduce noise and artifacts from the EEG signals [26]. Based on manual, trial-by-trial visual inspection of the data, the entire data sets of three patients were rejected due to excessive motion artifacts. For each subject, we recorded 30 trials for magno and parvo visual pathways and 34 trials for konio visual pathway. These signals were filtered (FIR, band-pass of 0.4–40 Hz). Channels in which the signal was a flat line for more than 5 seconds were removed. We then performed an independent component analysis [27] of all 32 channels and used the ICLabel algorithm [28] to identify artifacts (muscle, eye-movement, heart, line noise, and channel noise). Any component labeled as more than 70% artifact was removed from the data.

Next, we extracted data epochs starting 99 samples before and 512 samples after visual stimulus onset and subtracted the baseline (averaged over the 99 samples before stimulus) for each epoch and channel. From the remaining signals, we analyzed the 9 EEG channels corresponding to the occipital area (O1, O2, PO3, PO4, P3, P4, P7, P8, and Pz in the 10–20 system). As a final preprocessing step, we removed epochs that exceeded a threshold of ±80 μV and included only subjects with at least 20 trials for each stimulus. The preprocessing reduced the sample to 16 people living with MS (Table 1) and 16 controls, with on average 29, 29, and 32 trials for magno, parvo, and konio conditions, respectively.

### Visual evoked potentials

Visual evoked potentials (VEP) were determined (per subject, per pathway) by averaging over the nine occipital channels and all trials. The P100 latency was defined as the time at which the VEP reached its maximum within the window 75 to 120 ms after visual stimulus onset. Based on previous reports of VEP latencies in MS [13, 29], we extended the window to 120–160 ms after visual onset for the MS group. The accuracy of peak detection in this window was confirmed visually.

### Connectivity analysis

We used phase, and in particular, the Phase Locking Value (PLV), as a measure of synchrony because it reflects temporal relationships independent of signal amplitude [30]. For each electrode, we used the Hilbert transform to determine the phase of the filtered signal. For each pair of electrodes (k, l), we then determined their relative phase at each time point *t* and trial *tr*: *p*_*kl*_ (*t*, *tr*). Averaging this measure vectorially over all trials (TR) and time points (T) results in the average phase-locking value for each pair of electrodes [31] (with $j\;=\;\sqrt{-1}$):

$$PLV=\frac{1}{T}\frac{1}{TR}\left|\overset{T}{\underset{t=1}{\sum }}\overset{TR}{\underset{tr=1}{\sum }}e^{\left(j*p_{kl}\left(t,tr\right)\right)}\right|$$ (1)

**Fig 2:**
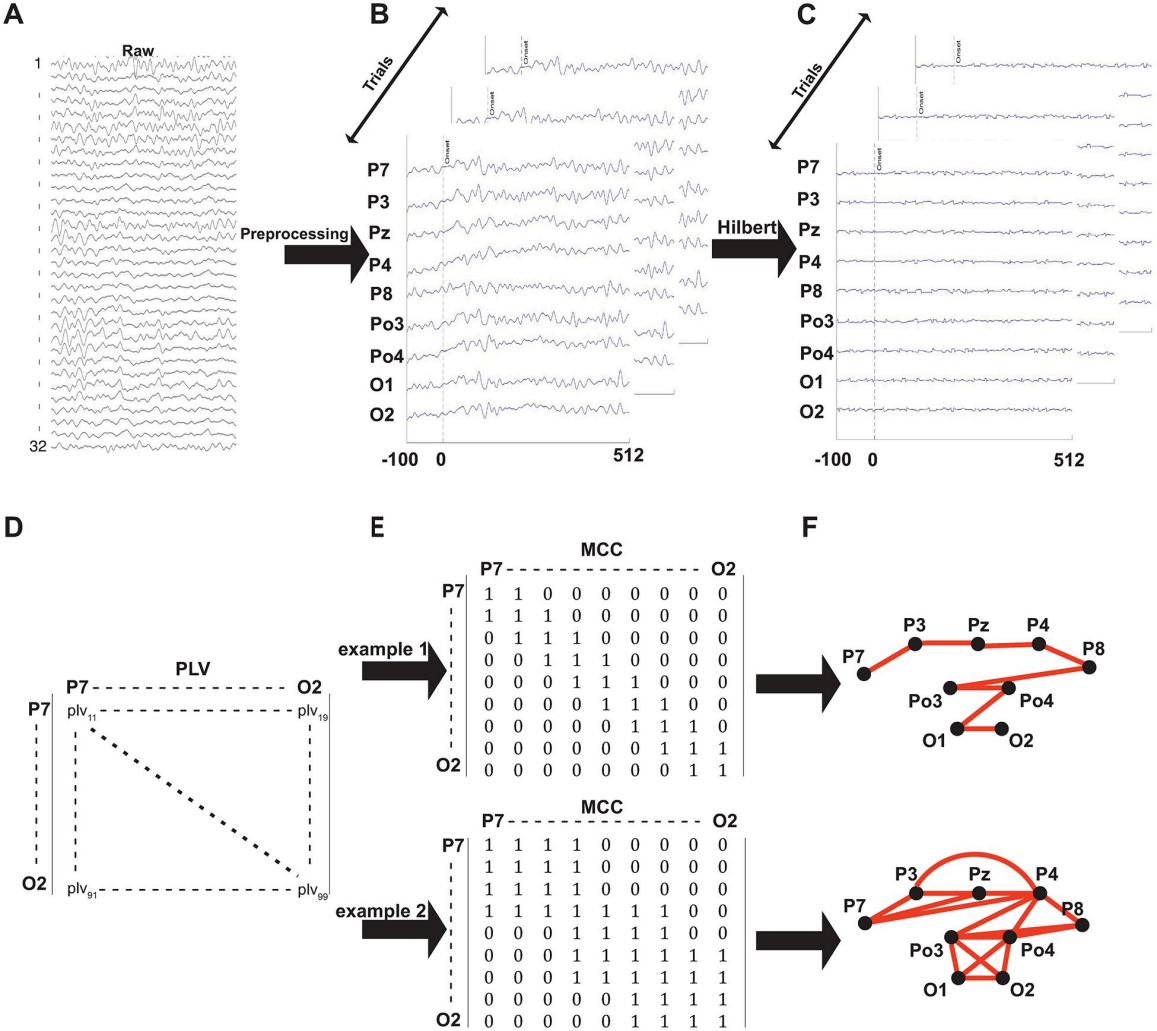
Constructing functional networks from EEG signals. (A) 32 channels of raw EEG data (B) Preprocessing results in the extraction of epochs from 99 samples before to 512 samples after stimulus onset for nine occipital channels (C) The Hilbert transform identifies the phase of the broadband signal for each epoch (D) We defined the strength of a functional connection between each pair of occipital electrodes as their Phase Locking Value (PLV) (E) From the PLV matrix, we construct the Minimally Connected Component (MCC). This panel shows two cartoon examples. In the top MCC, only neighbors are connected. In the bottom MCC, two sets of electrodes (P7, P3, Pz, P4) and (P8, Po3, Po4, O1, O2) are strongly connected to each other, with the groups only connected via P4. (F) Visual representation of the MCC example graphs. We use L and CC to quantify the graphs’ connectivity. In the top example, the distance from each node to another is relatively long (L = 3.34), and there is no clustering (CC = 0). In the bottom example, the average distance between each node is smaller (L = 1.69), and there is substantial clustering (CC = 0.82).

The PLV is zero if the signals in channels k and l are independent and tends to one for strongly coupled signals. Based on our choice of 9 occipital electrodes, PLV is a 9x9 matrix for each pathway and each subject and was calculated with the HERMES toolbox [32]. From these matrices, we extracted the Minimum Connected Component (MCC) (Fig 2). This algorithm starts with a graph in which all nodes (here electrodes) are included but without edges between them. Working through an ordered list of connectivity measures (PLV sorted large to small), binary connections are added to the graph until there is a path from each node to all the other nodes. The MCC, therefore, represents the minimal binary, undirected graph in which there is a path leading from each node to each other node; it identifies the most prominent connections in the network [33]. And, because connectivity here is based on a measure of synchrony (PLV), it identifies the electrodes that are most synchronized.

Two network metrics quantify functional integration and segregation characteristics of the MCC. The Clustering Coefficient (CC) reflects the extent to which nodes tend to cluster together; it is a measure of the cliquishness of neighborhoods (a local property). Suppose that a node j is directly connected to n other nodes (its neighbors in the graph); then at most *n*(*n − 1*)/*2* edges can exist between all of node j’s neighbors. Using C_j_ to refer to the fraction of these edges that exist for node j in the MCC, CC is the average of C_j_ over all nodes [34]. Watts and Strogatz given an intuitive explanation of CC in a friendship network; it quantifies the extent to which friends of a node are also friends of each other. For our data, this translates to the extent to which electrodes that are synchronized with a given electrode tend to synchronize with each other as well.

The characteristic path length (L) quantifies the typical separation between two nodes in the graph (a global property) and is sometimes used as a proxy for a network’s ability to transmit information rapidly. To calculate L, we first compute the distance d_k,l_ between nodes k and l in the graph. Because the connections in the MCC are binary, this equals the minimum number of edges one needs to traverse to go from k to l. The characteristic path length is the average of the shortest distance between all pairs of nodes in the network [35, 36]:

$$L=\frac{1}{N\left(N-1\right)}\underset{k,l}{\sum }d_{kl}$$ (2)

In our data, L represents a global measure of synchrony; if L is small, many occipital electrodes are synchronized with each other. If L is large, synchrony is limited to a small subset of electrode pairs.

Together, L and CC define the small-world nature of a network. Following Watts and Strogatz, we calculate small-worldness using the formula *σ* = *γ*/*λ* in which *γ = CC/CC*_*rand*_ and *λ = L/L*_*rand*_ where *CC*_*rand*_ and *L*_*rand*_ are the mean clustering coefficient and characteristic path length of the matched random networks, respectively [35, 36]. We estimated *CC*_*rand*_ and *L*_*rand*_ by averaging these measures over 100000 networks with random connectivity (but the same number of nodes and connections).

### Statistical analysis

We used linear mixed-effects models (fitlme in MATLAB (MathWorks, Inc., Natick, MA)) to determine the statistical significance of the main factors of the pathway (magno, parvo, konio), disease (MS, healthy), and their interaction as fixed effects. Each model included a random intercept per subject. In each case, we used a Kolmogorov-Smirnov test to compare the cumulative distribution of the residuals to the cumulative normal distribution with zero mean and a standard deviation equal to the standard deviation of the distribution of the residuals. None of the models had significantly non-normal residuals, hence the linear mixed model’s assumption of normality was warranted. After finding a significant main effect or interaction, we used linear contrasts (coefTest.m in MATLAB) to compare effects in pathways and used the false discovery rate method [37] to correct for multiple comparisons. In all cases, the threshold for significance was set at α = 0.05. We quantified effect size using Hedge’s g, which corrects for the upward bias in Cohen’s d for small sample sizes [38].

## Results

We used EEG to record scalp potentials evoked by visual stimuli designed to bias activity to the magno-, parvo-, or konio-cellular visual pathway. Participants viewed these stimuli passively. After preprocessing (Methods), our data set consisted of ~30 trials per condition for each of 16 people living with MS and 16 controls. We first present an analysis of visual latencies similar to previous work and, second, a novel graph-theoretic analysis of the synchrony of visually evoked potentials.

### Latency

**Fig 3:**
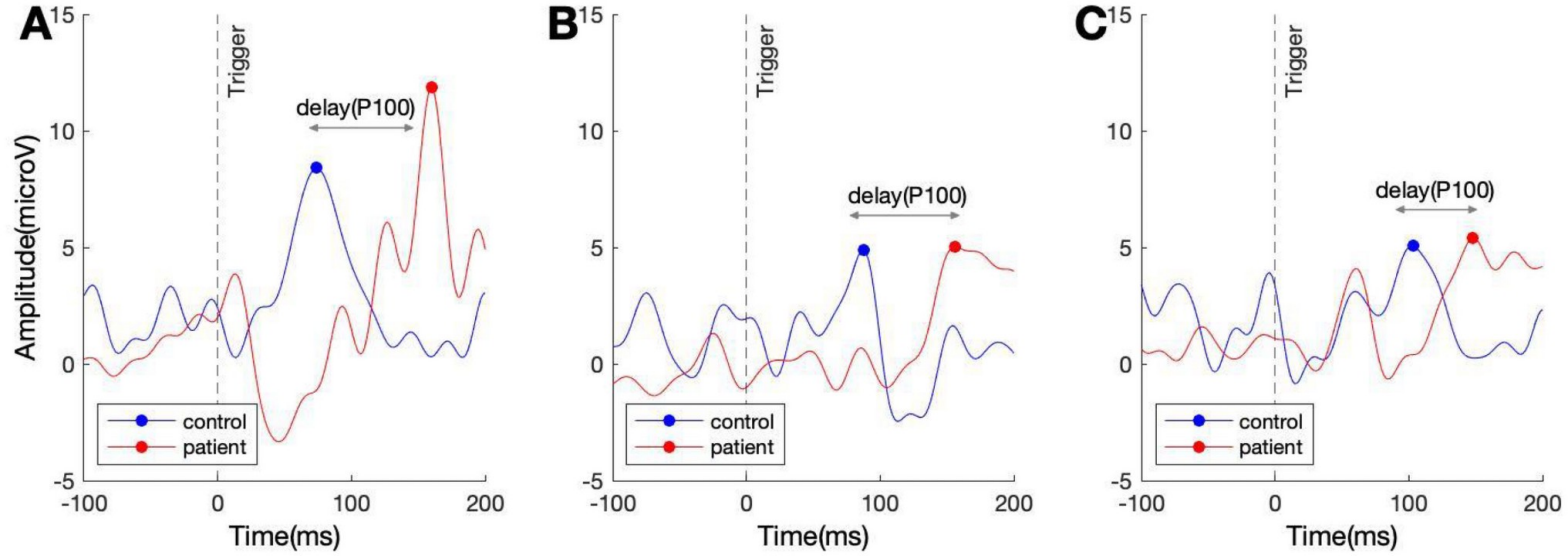
Example visual evoked potentials in a control (blue) and a patient (red). A) VEP for magno stimuli, B) VEP for parvo stimuli, and C) VEP for konio stimuli. The blue dots and the red dots demonstrate the estimated time point of the P100 in the controls and MS group, respectively. The pattern is seen here—long delays for each stimulus type, was consistent across patients (See main text).

We focused our latency analysis on the P100 of the VEP (Methods). The P100 in the MS group was similar in magnitude but substantially delayed compared to controls. Fig 3 shows example VEPs, averaged over all trials for one patient and one control. Across the group of controls, the average latency of the P100 was 94±17, 95±16, and 92±16 ms (mean ± standard deviation), for the magno, konio, and parvo stimuli, respectively. In the MS group, the corresponding latencies were 133±13, 137±14, 137±14 ms.

A linear mixed effects model showed a highly significant effect of disease (F (1,90) = 136.14, p = 1.05e-19) but no significant effect of pathway (F(2,90) = 0.40, p = 0.67), and no significant interaction between pathway and disease (F (2,90) = 0.51, p = 0.60).

As is evident from the large differences in mean latencies between the two groups and the small standard deviations within groups, the latency effects are highly robust. The corrected Cohen’s d effect sizes (aka Hedges’ g) associated with the magno, parvo, and konio stimuli were 2.45, 2.96, and 2.71, respectively. Hence, patients had substantially and significantly longer latencies, but given that there was no significant difference across pathways, the latency analysis lacks pathway specificity.

### Connectivity analysis

Next, we investigated synchrony among the nine occipital electrodes using a graph-theoretic approach (Methods). In brief, for each pair of electrodes, we extracted their phase-locking value and used this to define a 9x9 functional connectivity matrix (Fig 2D). We converted the matrix into a graph (the Minimally Connected Component; MCC; Fig 2E and 2F) in which each node represents an occipital electrode. Informally, the distance between two nodes in this graph represents the degree of synchrony between the signals of the corresponding electrodes. We characterize the connectivity of these graphs using the Characteristic Path Length (L) and the Clustering Coefficient (CC).

**Fig 4:**
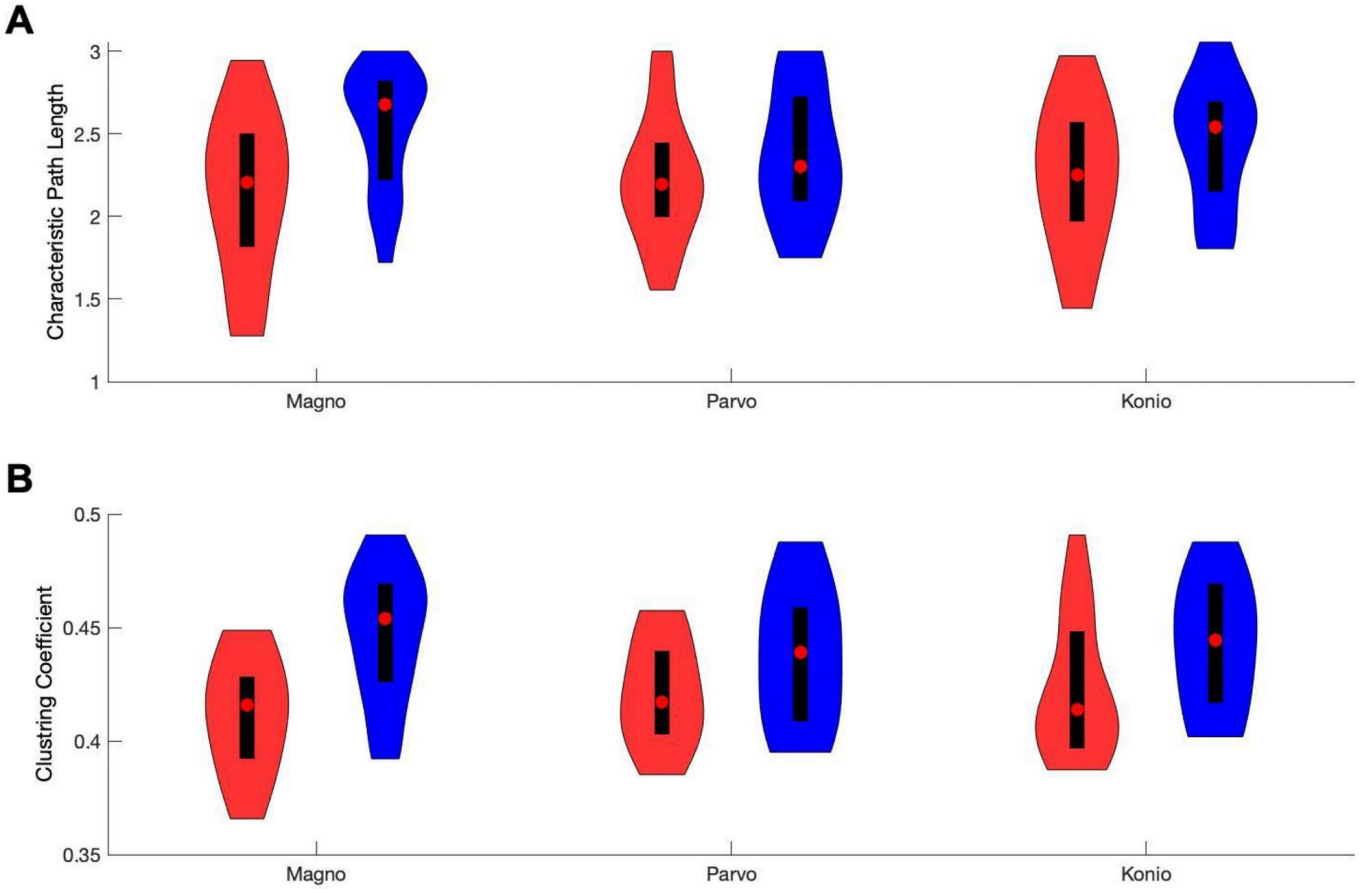
Distribution of connectivity measures for MS group and controls in three visual pathways. (A) Characteristic path length (L) for magno, parvo, and konio pathways. (B) Clustering coefficient (CC) for magno, parvo, and konio pathways. Red and blue violin plots show the distribution of the measurements in the MS group and controls, respectively. Red dots show the median, black bars the interquartile range. The figure illustrates that path length L and clustering coefficient CC were smaller in patients, specifically in the magnocellular pathway.

For the controls, the characteristic path length or typical distance between nodes (L) was 2.53±0.38, 2.37±0.41, 2.45±0.39, for the magno, parvo, and konio stimuli, respectively. In the patients, the corresponding L was 2.13±0.49, 2.22±0.39, 2.25±0.44. A linear mixed effects model showed that L was significantly smaller in MS patients (main effect of disease: F (1,90) = 4.45, p = 0.037)). There was neither a significant interaction between disease and pathway (F(1,90) = 0.30, p = 0.74), nor a main effect of pathway (F (1,90) = 1.79, p = 0.17). A more specific analysis (using linear contrasts) of the effects in the three pathways demonstrated that the decrease in L was attributable to magnocellular stimuli (F (1, 90) = 7.806, p = 0.006), with non-significant differences for the parvo and konio stimuli (F (1, 90) < 1.92, p> 0.2). The effect sizes (Hedge’s g) for L were 0.89, 0.37, and 0.47 for magno, parvo, and konio, respectively. Fig 4A compares the distribution of L between patients and controls for each of the stimulus types.

The Clustering Coefficient (CC) provides a complementary view of the graph by quantifying the tendency of nodes to cluster in triplets. A CC near 0 corresponds to a network in which strong synchrony of three electrodes is rare, while a CC of 1 is a network in which all triplets of electrodes are strongly synchronized. The CC for the magno, parvo, and konio stimuli was 0.45±0.03, 0.44±0.03, 0.44±0.03 in the controls, and 0.41±0.03, 0.42±0.02, 0.42±0.03 in the MS group. The statistical analysis revealed a significant main effect of disease (F (1,90) = 11.07, p = 0.001), but no interaction between pathway and disease (F (1,90) = 0.15, p = 0.86), and no main effect of pathway (F (1,90) = 1.76, p = 0.18). The linear contrast analysis of the three pathways showed a significant decrease of CC in the magnocellular visual pathway (F (1, 90) = 13.7, p = 0.0004), with non-significant effects for the parvo and konio stimuli (F (1,90) < 2.77, p> 0.06). Hedge’s g for CC was 1.26, 0.58, and 0.61 for magno parvo, and konio stimuli, respectively. Fig 4B compares the distribution of CC between patients and controls for each of the stimulus types.

**Fig 5:**
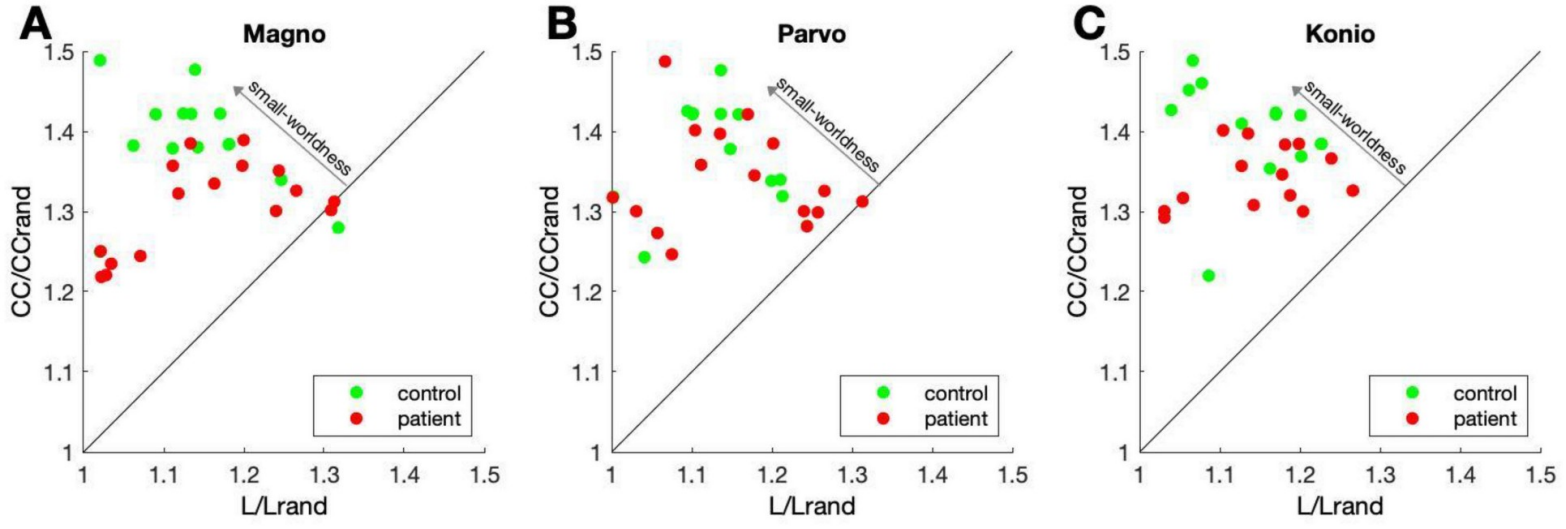
Small-worldness of occipital networks. Each data point shows normalized clustering coefficient (*CC*/*CC*_*rand*_; y-axis) and the normalized path length (*L*/*L*_*rand*_: x-axis) for each patient (red dots) and controls (green dots). The ratio of the measures on the y and x-axis is *σ*, which defines the small-worldness of the network. Data points further away from the slope 1 line (*σ* = 1) represent networks with increased small-worldness. A) Magnocellular pathway. B) Parvocellular pathway. C) Koniocellular pathway. The figure illustrates that small-worldness was smaller in patients, specifically in the magnocellular pathway.

These local (CC) and global (L) measures of connectivity can be combined into a single number that reflects the small-world nature of the network. A small-world network typically has strong local clustering and a path length that is not longer than the average path length of a network with the same number of nodes but random connections. Following [36], we quantified small-worldness using *σ*: the ratio of CC and L, normalized by the CC and L of random networks (Methods). This quantity, *σ*, is larger than 1 for small-world networks. For controls, *σ* for magno, parvo, and konio stimuli was 1.24±0.11, 1.24±0.12, and 1.25±0.10, respectively. In the patients, *σ* was 1.14±0.07, 1.17±0.12, and 1.17±0.08, respectively. In other words, the occipital networks in the MS group had reduced small-worldness, particularly for magnocellular stimuli. Fig 5 illustrates this; whereas the red (patient) and green (controls) data points are intermixed, particularly for the parvocellular pathway (panel B), the red dots (patients) are closer to the *σ* = 1 line than the green dots (controls) in the magnocellular pathway (panel A).

## Discussion

We investigated the influence of MS on visual processing. Using visual stimuli to bias activity to the magno, parvo, or konio cellular pathways, we confirmed previous findings that visual evoked potentials were substantially delayed in the MS group. These delays affected all three pathways, and we found no significant differences among pathways. We then quantified the synchrony of EEG signals across occipital electrodes using the graph-theoretic concept of small-worldness and found that MS reduced small-worldness significantly in the magnocellular pathway, but not the parvo or koniocellular pathway. In this section, we first discuss the limitations of our study and then provide an outlook for future studies investigating the underlying neural changes and potential clinical usage.

### Limitations and future directions

The number of participants in our study was relatively small (16 patients, 16 controls). That implies we could detect only robust effects. For VEP latency effects, this robustness has been established before with Hedge’s g effect sizes also above 2 [28, 38], and our study shows that changes in synchrony for magnocellular stimuli are smaller but robust nevertheless (effect sizes ~0.89 for L and 1.26 for CC). Small sample sizes, however, imply that the absence of evidence (e.g., no significant change in synchrony for parvo or konio stimuli) should be interpreted with care; significant differences in connectivity may be found with larger sample sizes. This, however, does not affect our main conclusion that MS has the largest effect on synchrony in the magnocellular pathway. Another limitation was that, given the limited sample size, our study necessarily treated the entire patient sample as a single group, and we cannot determine reliably whether specific aspects of the disease (e.g., time since diagnosis) affect synchrony.

We chose the three stimuli to bias activity towards different pathways. This bias is likely incomplete, as no visual stimulus can fully isolate a pathway [17, 39]. The fact that our magno-stimuli also drive the other two pathways (albeit to a lesser extent) means that the differences we found are a lower bound on the differences between the pathways. In future work, it may be beneficial to design stimulus sets that increase pathway targeting specificity. That said, the simplicity of our stimulus set is appealing, and any increase in complexity could reduce applicability as a tool in a clinical/diagnostic setting. We also note that it is important to choose visual stimuli that evoke similar responses in each of the pathways (i.e., no main effect of the pathway). Otherwise, pathway-specific differences could be attributed to the overall drive provided to each pathway. The absence of significant main effects of the pathway in each of our measures shows that this did not play a major role in our findings.

The seven degree stimulus limits the fraction of the visual pathway that is probed to probably less than 25% of the retinal ganglion cells [40, 41]. Moreover, in the cortex, such a single stimulus can lead to a reduction of the overall signal strength due to source averaging. Multifocal visual evoked potentials (mfVEP) [42] could address this limitation; it allows many visual field locations to be probed independently and simultaneously and may be able to further differentiate damage of different pathways in MS [43].

### Synchrony

One might have expected that demyelination would reduce both global and local measures of synchrony. Contrary to this, we found an increase in long range synchrony in the MS group (a smaller L) but a decrease in short range synchrony (a smaller CC). In future work, it would be interesting to determine the generality of this finding, for instance, by examining synchrony between even more widely spaced electrodes or in a behavioral paradigm that is not specifically targeted to the visual system (e.g., multimodal sensory stimulation or resting stage EEG). Nevertheless, for the data at hand, short-range synchrony decreased more than long-range synchrony increased, and hence the occipital network in the MS group had reduced small-worldness (the ratio of CC and L).

In a small-world network, nearby nodes are likely to be connected (i.e., synchronous), and even remote nodes can be reached in few steps [44]. This implies that information spreads rapidly throughout the network; it is a measure of efficiency. Watts and Strogatz (1998) illustrate this with an infectious disease that spreads much more rapidly and easily in a physical world with small-world connectivity than in a world with random connections. In neural processing, the spread of information through the network is likely necessary for the integration of many sources of information. We speculate that visual deficits in the MS group could be related to the reduced small-worldness of their visual network. The predominance of visual deficits when using stimuli that provide the strongest drive to the magnocellular pathway (see Introduction), together with our finding that small-worldness is significantly reduced only in the magnocellular network, supports this view. However, a more direct test of this hypothesis would require recording EEG while subjects perform a battery of visual tests and determining whether small-worldness is correlated with behavioral performance across subjects or longitudinally in the same subjects as the disease progresses. In this context, it would also be interesting to extend the analysis to higher level cognition and relate global connectivity measures with cognitive performance, as has been done in patients with schizophrenia [45].

### Neural origins

On their own, EEG measures cannot pinpoint the precise neural origins of a deficit. Previous work, however, has used diffusion tensor imaging (DTI) and optical coherence tomography (OCT) to argue that structural changes in the retina, optic nerve, optic tract, and optic radiation all contribute to the increased latency of visually evoked responses in MS [46–48]. While synchrony likely also depends on structural changes at these early levels of the visual hierarchy, the fact that visual synchrony varies with arousal and top-down attention [49] suggests that it may also be sensitive to structural changes at the cortical level. This could be investigated by combing DTI and EEG recordings, ideally using tasks that are known to modulate cortical synchrony [49].

### Biomarkers

Although we found a substantial effect size (g>0.8) for MS induced changes in magnocellular synchrony, this was dwarfed by the effect size for latency (g >2 in our sample). This suggests that latency is better suited for diagnosis than our measures of synchrony. Beyond diagnosis, however, the relatively low cost of obtaining multi-channel EEG, together with the exponential growth in computing power that enables the multi-channel analyses, provide an opportunity to learn more about the changes in neural processing in MS.

Moreover, previous work has identified other candidate biomarkers based on EEG. For instance, global alpha power [50], frontal theta/beta ratio [50], and inter-hemispheric mutual information [20], information transfer efficiency and modularity [51], as well as resting state functional connectivity [19, 52, 53] are affected by MS. In principle, such measures, by providing complementary views of neural processing in MS, could be combined into a more powerful diagnostic tool. Investigating such applications, however, requires larger scale, and longitudinal studies to answer the important and open empirical question which of these measures may allow differential diagnosis of MS [54], predict disease progression [55], or document the efficacy of patient treatment [56].
